# DMSO potentiates the suppressive effect of dronabinol on sleep apnea and REM sleep in rats

**DOI:** 10.1186/s42238-023-00199-4

**Published:** 2023-07-28

**Authors:** Michael W. Calik, David W. Carley

**Affiliations:** 1 Center for Sleep and Health Research, University of Illinois Chicago, Chicago, IL USA; 2 Department of Biobehavioral Nursing Science, University of Illinois Chicago, Chicago, IL USA; 3 Department of Biobehavioral Nursing Science, College of Nursing, University of Illinois Chicago, 845 South Damen Avenue (M/C 802), Room 740, IL 60612 Chicago, USA; 4 Department of Medicine, University of Illinois Chicago, Chicago, IL USA

**Keywords:** DMSO, Cannabinoids, Dronabinol, Sleep apnea, REM sleep

## Abstract

**Introduction:**

Dimethyl sulfoxide (DMSO) is an amphipathic molecule with innate biological activity that also is used to dissolve both polar and nonpolar compounds in preclinical and clinical studies. Recent investigations of dronabinol, a cannabinoid, dissolved in DMSO demonstrated decreased sleep apnea frequency and time spent in REM sleep in rats. Here, we tested the effects of dronabinol dissolved in 25% DMSO diluted in phosphate-buffered saline (PBS) to rule out potentiating effects of DMSO.

**Methods:**

Sprague–Dawley rats were anesthetized and implanted with bilateral stainless steel screws into the skull for electroencephalogram recording and bilateral wire electrodes into the nuchal muscles for electromyogram recording. Each animal was recorded by polysomnography. The study was a fully nested, repeated measures crossover design, such that each rat was recorded following each of 8 intraperitoneal injections separated by three days: vehicle (25% DMSO/PBS); vehicle and CB_1_ antagonist (AM 251); vehicle and CB_2_ antagonist (AM 630); vehicle and CB_1_/CB_2_ antagonist; dronabinol (CB_1_/CB_2_ agonist); dronabinol and CB_1_ antagonist; dronabinol and CB_2_ antagonist; and dronabinol and CB_1_/CB_2_ antagonists. Sleep was manually scored into NREM and REM stages, and sleep apneas were quantified.

**Results:**

Dronabinol dissolved in 25% DMSO did not suppress sleep apneas or modify sleep efficiency compared to vehicle controls, in contrast to previously published results. However, dronabinol did suppress REM sleep, which is in line with previously published results.

**Conclusions:**

Dronabinol in 25% DMSO partially potentiated dronabinol’s effects, suggesting a concomitant biological effect of DMSO on breathing during sleep.

## Introduction

Dimethyl sulfoxide (DMSO; (CH_3_)_2_SO) is an amphipathic molecule used to dissolve both polar and nonpolar compounds in preclinical and clinical studies (Jacob and Torre 2009; Santos et al. 2003). Moreover, DMSO is known to increase the bioavailability of lipophilic drugs (Watanabe et al. 2000; Brayton 1986; Elzinga et al. 1989) and is widely distributed throughout the body, including the brain (Denko et al. 1967; Hucker et al. 1966).

Although most often used as a solvent, existing evidence convincingly demonstrates that DMSO has innate biological activity that may confound experimental results when it is used as a solvent for drug delivery. For example, DMSO is known to decrease the integrity of the blood–brain barrier (BBB) (Broadwell et al. 1982), block fast axonal transport in the vagus nerve (Donoso et al. 1977), and modulate morphine-induced antinociception (Fossum et al. 2008). Further, DMSO induces hypothermia (Julien et al. 2012), reduces pulmonary ventilation (Takeda et al. 2016), enhances hippocampal-dependent spatial memory accuracy, exerts anxiogenic (Penazzi et al. 2017) and antiepileptic (Carletti et al. 2013) effects, and changes sleep architecture (Cavas et al. 2005). DMSO has also been shown to decrease the occurrence of spontaneous type 1 diabetes by modulating the autoimmune response (Lin et al. 2015). DMSO also increases cell permeability and is known to be cytotoxic (Galvao et al. 2014; Notman et al. 2006).

Proper delivery of cannabinoids necessitates dissolving cannabinoids in amphipathic solvents (Momenzadeh et al. 2023). Previously, we have shown that administration of dronabinol, a synthetic cannabinoid type 1 (CB_1_) and cannabinoid type 2 (CB_2_) receptor agonist, dissolved in undiluted DMSO decreased sleep apnea index and rapid eye movement (REM) sleep in rats (Carley et al. 2002; Calik and Carley 2017). Recent experiments using a model of reflex apnea in anesthetized rats implicates the activation of cannabinoid (CB) receptors on the nodose ganglia of vagus nerves in the apnea-suppressive effect (Calik and Carley 2014; Calik et al. 2014), with little impact deriving from CB receptors located in the brain (Calik and Carley 2016). In contrast, dronabinol’s effects on REM sleep occurs via activation of CB_1_ receptors in the brain (Goonawardena et al. 2015; Navarro et al. 2003; Silvani et al. 2014). Considering DMSO’s pleotropic effects, these studies could not distinguish between the effects of cannabinoids from those of DMSO. Moreover, it is unknown if these effects of dronabinol were partially potentiated by the biologically active solvent, DMSO. Here, we report that sleep apneas were not suppressed, but REM sleep was suppressed, by dronabinol dissolved in 25% DMSO.

## Materials and methods

### Animals

Adult male Sprague–Dawley rats (*n* = 12; ~ 275 g) purchased from Harlan Laboratories (Indianapolis, IN, USA) were initially housed in duplicate, maintained on a 12:12 h light:dark cycle (lights on 8:00 am, lights off 8:00 pm) at 22 ± 0.5 °C, and allowed ad libitum access to food and water. After surgery, rats were housed singly to prevent loss of headsets. All animal procedures and protocols were approved by the Institutional Animal Care and Use Committee of the University of Illinois at Chicago.

### Surgical procedures

Implantation of polygraphic headsets has been described before (Carley et al. 2002; Calik and Carley 2017). Rats were anesthetized (ketamine:xylazine 100:10 mg/kg; buprenorphine 0.1 mg/kg), stereotaxically immobilized, and implanted with electroencephalographic (EEG) screw electrodes bilaterally threaded into the frontal and parietal bones. Electromyographic (EMG) wire electrodes were implanted in the dorsal nuchal musculature and tunneled subcutaneously to the skull. EEG and EMG leads were soldered to a miniature plastic connector plug (i.e. headset) and affixed to the skull acrylic dental cement. Scalp wounds were closed with Vetbond Tissue Adhesive. Rats were allowed to recover for 7 days before beginning a week of acclimation to handling and plethysmographic recording chambers.

### Plethysmography, polysomnography and treatment protocol

Polysomnography (PSG) procedures have been previously described (Calik and Carley 2017). Rats underwent nine 6-h PSG recordings, separated by at least 3 days. All recording sessions began at 10:00 and continued until 16:00. Each rat received an IP injection at 09:45. Rats were immediately placed inside a bias-flow-ventilated (2 l/min) whole-body plethysmograph (PLYUNIR/U, Buxco Electronics, Wilmington, DE, USA). A flexible cable was inserted through a narrow “chimney” into the main plethysmography chamber and attached to the rat’s headset. Rats underwent a week of acclimation to handling and to plethysmographic recording chambers, including being connected to the flexible cable. After acclimation, rats were recorded for 6 h for one occasion prior to the first experimental session to permit adaptation to the recording system, and to assess the quality of EEG and EMG signals. If signal quality was good, then the rats underwent a repeated measures random order crossover design, such that each rat received each of 8 IP injections exactly one time in random order: vehicle alone (25% DMSO in PBS; 1 ml); dronabinol alone (10.0 mg/kg; Mylan Pharmaceuticals, Morgantown, WV); AM251 alone (5.0 mg/kg, Tocris Bioscience, Bristol, UK); AM630 alone (5.0 mg/kg, Tocris Bioscience); or AM251/630 combination (5.0/5.0 mg/kg); or a combination injection (dronabinol/AM251 or dronabinol/AM630 or dronabinol/AM251/AM630). All drugs were dissolved in 25% DMSO in PBS. Drug doses were based on previous studies (Calik and Carley 2017; Bisogno et al. 2009; Mallet et al. 2008). Respiratory signals from whole body plethysmography were amplified, band-passed filtered (1 to 10 Hz; CyberAmp 380, Axon Instruments, Sunnyvale, CA), and digitized (250 samples/s; Biologic Sleepscan Premier, Natus, San Carlos, CA). EEG and EMG signals were amplified and band-passed filtered (0.5 to 100 Hz and 10 to 100 Hz, respectively) and digitized (250 samples/s; Bio-logic Sleepscan Premier).

Visual sleep scoring was conducted by a blinded and experienced technician. Sleep stages (wake, NREM, and REM) were scored for every 30-s epoch of the 6-h recording. Wakefulness was characterized by high-frequency and low-amplitude (beta/alpha waves) EEG with high EMG tone. NREM sleep was characterized low-frequency and high-amplitude (delta waves) and low EMG tone, while REM sleep was characterized by high-frequency and high-amplitude (theta waves) EEG and an absence of EMG tone. Sleep stage percentages, defined as total time spent in a specific sleep stage (awake, NREM, or REM) divided by total time in the plethysmograph, and sleep efficiency, defined as total time spent in sleep (both NREM and REM) divided by total time spent in the plethysmograph, were also quantified.

Digitized apneas from whole body plethysmography were visualized and scored by a blinded and experienced technician using Bio-logic Sleepscan Premier. Respiration was marked peak-to-peak (breath duration in seconds), and sleep apneas (apneas only occurring during) were scored as a cessation of breathing for at least 2 s, and were quantified as an sleep apnea index (apneas/hour) and separately stratified for overall sleep and NREM sleep. Due to a small amount of time, or no time, spent in REM sleep, a REM sleep apnea index was not calculated because there would be low estimation precision and many rats would have a “null” data point for REM apnea index (Calik and Carley 2017). Sleep apneas were further subdivided into post-sigh (preceded by a breath at least 50% larger than the average of the preceding 5 breaths) sleep and spontaneous sleep apneas (not preceded by an augmented breath) and shown as post-sigh and spontaneous sleep apnea indices, respectively (Ramirez et al. 2013; Saponjic et al. 2007).

### Statistical analysis

Data (mean ± SEM) were analyzed using IBM SPSS Statistics 22 (New York, NY) linear mixed model analysis using treatment (CB agonist, CB antagonist, and CB agonist/antagonist interaction) as a fixed effect and animal as a repeated measure, followed by post hoc multiple comparison tests with Sidak’s correction if there were significant main effects or a significant interaction of main effects. Repeated covariance structure was chosen according to the best-fit Schwarz’s Bayesian information criterion (Wang and Goonewardene 2004).

## Results

**Fig. 1:**
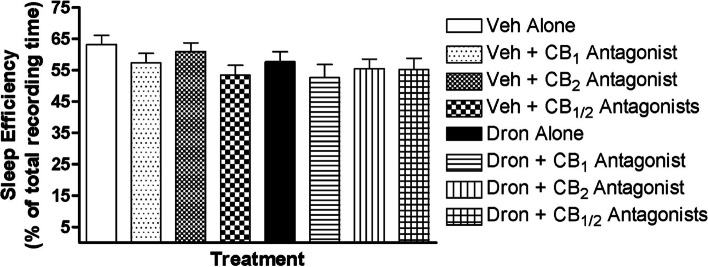
Sleep efficiency quantified as a percentage of time spent asleep from 6 h recordings of conscious chronically-instrumented rat experiments. Vehicle (25% DMSO in PBS) or dronabinol (10 mg/kg) was injected IP in combination with vehicle or CB_1_ receptor (AM 251, 5 mg/kg) or CB_2_ receptor (AM 630, 5 mg/kg) antagonist, or both. There were no significant main effects. Data (mean ± SEM) were analyzed using linear mixed model analysis with repeated/fixed measures (CB agonist and CB antagonist)

**Fig. 2:**
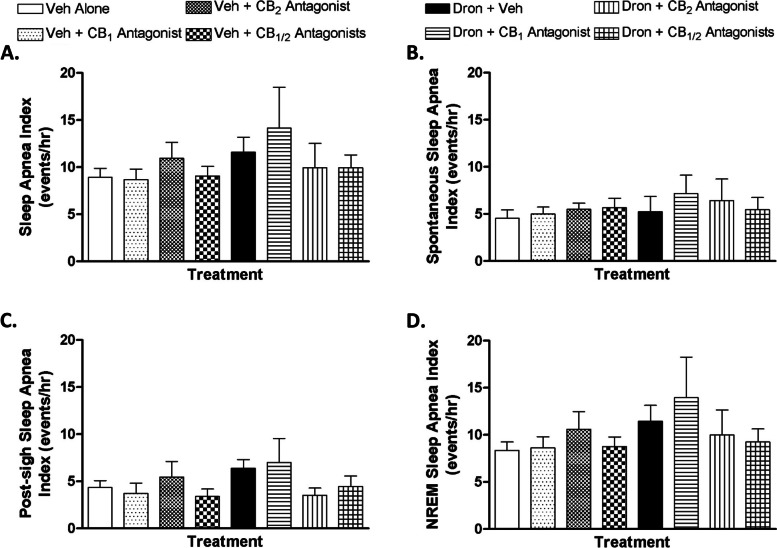
Sleep apnea (**A**), spontaneous sleep apnea (**B**), post-sigh sleep apnea (**C**) and NREM sleep apnea (**D**) indices quantified from 6 h recordings of conscious chronically-instrumented rat experiments. Indices were quantified as events/hr. Vehicle (25% DMSO in PBS) or dronabinol (10 mg/kg) was injected IP in combination with vehicle (solid bars) or CB_1_ receptor (AM 251, 5 mg/kg) or CB_2_ receptor (AM 630, 5 mg/kg) antagonist, or both. There were no significant main effects. Data (mean ± SEM) were analyzed using linear mixed model analysis with repeated/fixed measures (CB agonist and CB antagonist)

**Fig. 3:**
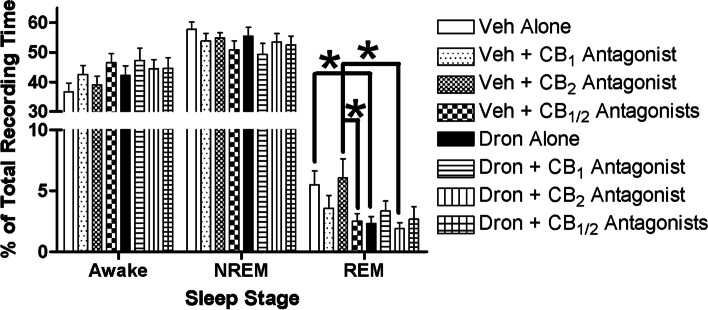
Awake time (left), and NREM (center) and REM (right) sleep as a percentage of total recording time quantified from 6 h recordings of conscious chronically-instrumented rat experiments.. Vehicle (25% DMSO in PBS) or dronabinol (10 mg/kg) was injected IP in combination with vehicle or CB_1_ receptor (AM 251, 5 mg/kg) or CB_2_ receptor (AM 630, 5 mg/kg) antagonist, or both. Dronabinol and a combination of dronabinol and CB_2_ antagonist significantly reduced REM sleep. Data (mean ± SEM) were analyzed using linear mixed model analysis with repeated/fixed measures (CB agonist and CB antagonist) followed by post hoc multiple comparison tests with Sidak’s correction if there were significant main effects or a significant interaction of main effects. *p < 0.05

Rats (*N* = 12) were injected with a CB receptor agonist (dronabinol) or vehicle, and with CB_1_/CB_2_ receptor antagonists (AM251, AM630, or both) or vehicle. Sleep efficiency is depicted in Fig. 1. Stratified sleep apnea indexes are presented in Fig. 2, and time spent in wakefulness, NREM or REM sleep is shown in Fig. 3.

The linear mixed model analysis revealed a significant effect of antagonist treatment (*F*_3, 64.41_ = 2.86, *p* = 0.04) on sleep efficiency (Fig. 1); however, post hoc analysis revealed no significant differences among the antagonist treatment groups (*p* > 0.05). There were no significant main effects (*p* > 0.05) on overall sleep apnea index (Fig. 2A), spontaneous sleep apnea index (Fig. 2B), post-sigh sleep apnea index (Fig. 2C), and NREM sleep apnea index (Fig. 2D). These results are in opposition with previous research showing an effect of dronabinol in 100% DMSO (1 ml) on sleep efficiency and sleep apnea frequency (Carley et al. 2002; Calik and Carley 2017).

No treatment effects were observed for time spent in NREM sleep (Fig. 3). Antagonist treatment had an effect (*F*_3, 65.43_ = 2.84, *p* < 0.05) on time spent awake, but post hoc analysis revealed no differences among the antagonist treatments (*p* > 0.05). There was significant agonist/antagonist interaction (*F*_3, 77.00_ = 3.68, *p* = 0.02) observed for REM sleep time. Post hoc analysis revealed that rats receiving dronabinol alone (2.31 ± 0.58%, *N* = 12) or dronabinol and CB_2_ antagonist (2.68 ± 1.02%, N = 12) had significantly (*p* < 0.01) decreased REM sleep compared to vehicle only (5.49 ± 1.13%, *N* = 12) or CB_2_ antagonist only (6.06 ± 1.56%, *N* = 12), respectively. In addition, there was significant difference between CB_2_ antagonist only (6.06 ± 1.56%, *N* = 12) and CB_1_/CB_2_ only (2.53 ± 0.62%, *N* = 12). These results are similar with previous research (Carley et al. 2002; Calik and Carley 2017).

## Discussion

The major findings of the present study are: (a) dronabinol in 25% DMSO failed to suppress sleep apneas or to decrease sleep efficiency (Figs. 1 and 2); and (B) dronabinol in 25% DMSO decreased REM sleep (Fig. 3), which is in contrast and in line, respectively, with previously reported results (Carley et al. 2002; Calik and Carley 2017). The only difference in experimental protocol between this study and our previously reported studies was the concentration of DMSO used to dissolve dronabinol.

Dronabinol, a synthetic version of Δ9-THC, is a lipophilic substance that has been previously used to suppress sleep apneas in preclinical studies (Carley et al. 2002; Calik and Carley 2017) by a mechanism that involves modulation of vagus nerve activity via CB receptors on nodose ganglia (Calik and Carley 2014; Calik et al. 2014). In those preclinical studies, dronabinol was dissolved in undiluted DMSO since DMSO was known to increase bioavailability of the lipophilic drugs (Watanabe et al. 2000; Brayton 1986; Elzinga et al. 1989). Although undiluted DMSO alone did not alter sleep apnea expression in these studies (Brayton 1986; Broadwell et al. 1982), DMSO may have altered the effects of dronabinol, since it is known to block fast axonal transport in the vagus nerve. Still, it is important to note that dronabinol dissolved in sesame oil rather than DMSO did reduce sleep apnea frequency in two clinical trials in patients with obstructive sleep apnea syndrome (Carley et al. 2018; Prasad et al. 2013).

In rats, dronabinol impacted sleep efficiency and sleep apnea expression only when dissolved in 100% DMSO (Carley et al. 2002; Calik and Carley 2017). Decreasing the concentration of DMSO, as in the present study, eliminated the sleep apnea suppressive effects. However, vehicle controls in those previous studies and in the present study had similar sleep efficiencies and sleep apnea indices, arguing against the effect of DMSO alone on these parameters. The simplest explanation for this effect was that the increased concentration of DMSO increased the absorption and bioavailability of dronabinol (Watanabe et al. 2000). Increased absorption and bioavailability of dronabinol could increase activation of CB receptors on the nodose ganglia, which play a part in sleep apnea suppression (Calik and Carley 2014; Calik et al. 2014). However, we cannot rule out that DMSO potentiated the effects of dronabinol, by either modulating vagal nerve activity (Donoso et al. 1977; Sams et al. 1966), or by modulating pulmonary ventilation (Takeda et al. 2016), or both. Future studies using other solvents, like propylene glycol or kolliphor, can rule out potentiating effects of DMSO (Momenzadeh et al. 2023). Another plausible explanation is that dronabinol had increased access to the brain because DMSO decreased the integrity of the BBB (Broadwell et al. 1982). The BBB is efficient at limiting the transport of Δ9-THC into the brain (Nahas et al. 2002), thus decreased BBB integrity may increase the amount of dronabinol available to the brain and thus, modulation of breathing via centrally-located CB receptors (Pertwee 2005). However, a recent study that injected dronabinol into the brain demonstrated no effect on reflex apneas in anesthetized rats (Calik and Carley 2016).

In contrast to the effects on sleep apnea and sleep efficiency, the only measured effect of dronabinol in 25% DMSO was reduced REM sleep, which is in line with previous work using 100% DMSO (Carley et al. 2002; Calik and Carley 2017). Although the amount of REM sleep in vehicle treated rats was similar for 25% and 100% DMSO, the effects of dronabinol were larger in the 100% DMSO formulation compared to the 25% DMSO. This argues that 100% DMSO increased the bioavailability of dronabinol in comparison to 25% DMSO, since CB receptors located in the brain play a role in sleep regulation in rats (Silvani et al. 2014), though both DMSO formulations allowed for enough dronabinol to be available to decrease REM sleep. Vagal nerve activity also has been implicated in sleep regulation (Rizzo et al. 2003; Valdes-Cruz et al. 2008), so the combination of dronabinol and 100% DMSO, with their known effects on the vagus nerve as previously discussed, might decrease REM sleep to a greater extent. CB_1_ and CB_1_/CB_2_ antagonism decreased REM sleep in rats treated with vehicle but failed to attenuate dronabinol-induced decreases in REM sleep, similar to previously reported results (Calik and Carley 2017; Goonawardena et al. 2015). AM 251 and AM 630 concentrations were chosen based on previous reports of these compounds blocking the antinociceptive properties of endocannbinoids. (Bisogno et al. 2009; Mallet et al. 2008). Both antagonists are known to cross the blood–brain barrier (Hodge et al. 2008; Guidali et al. 2011; Gatley et al. 1996; Chin et al. 2008). It is unknown why the differential effects of CB antagonism in vehicle or dronabinol treated rats, but it has been hypothesized that CB_1_ antagonism-induced decreases in REM sleep may be caused by inhibition of CB_1_-dependent modulation of GABAergic activity in sleep-relevant centers of the brain (Calik and Carley 2017; Goonawardena et al. 2015).A limitation of this study is the use of male rats only. Previous studies have shown sex difference in rodent models of sleep and sleep apnea. Future studies using female rats will be needed to confirm the effects of dronabinol and DMSO (Dib et al. 2021; Boukari et al. 2016).

In conclusion, we show that dronabinol, a non-specific cannabinoid receptor agonist shown to suppress sleep apneas and REM sleep, does not suppress sleep apneas in 25% DMSO vehicle. This adds to the growing literature that DMSO is not simply a compound used to dissolve polar and nonpolar compounds but is a compound with its own innate biological activity.

## Data Availability

The datasets used and/or analyzed during the current study are available from the corresponding author on reasonable request.
